# ﻿Discovery of the termitophilous genus *Trichopsenius* Horn, 1877 (Coleoptera, Staphylinidae, Aleocharinae) from China with description of a new species

**DOI:** 10.3897/zookeys.1152.99290

**Published:** 2023-03-06

**Authors:** Ri-Xin Jiang, Xiu-Dong Huang, Xiang-Sheng Chen

**Affiliations:** 1 Institute of Entomology, Guizhou University, Guiyang 550025, Guizhou, China; 2 The Provincial Special Key Laboratory for Development and Utilization of Insect Resources of Guizhou, Guizhou University, Guiyang, 550025, Guizhou, China; 3 The Provincial Key Laboratory for Agricultural Pest Management of Mountainous Region, Guiyang 550025, Guizhou, China

**Keywords:** Guizhou, rove beetles, termites, termitophily, Trichopseniini

## Abstract

The termitophilous genus *Trichopsenius* Horn, 1877 is recorded from China for the first time. A new species, *Trichopseniushuaxiensis***sp. nov.** is described; it was collected in a nest of the termite genus *Reticulitermes* Holmgren from a dead and flattened pine tree.

## ﻿Introduction

The rove beetle genus *Trichopsenius* Horn, 1877 (Aleocharinae, Trichopseniini) is composed of 12 known species, and discontinuously distributed from Europe (one species from Spain; Kistner and Assing 1995) and North Africa (one species; Kanao and Maruyama 2019) to North America (five species; LeConte 1863; Seevers 1941, 1945, 1957; Pasteels and Kistner 1971). In Asia, five species are known from Japan, but there are not any records outside of Japan (Naomi and Terayama 1986, 1996).

All known members of the genus *Trichopsenius* are termitophiles and associated with the termite genus *Reticulitermes* Holmgren (Blattodea, Rhinotermitidae; Kanao and Maruyama 2019). Immature stages of *Trichopsenius* are known only for two American species, *T.depressus* (LeConte, 1863) and *T.frosti* Seevers, 1945 (Howard and Kistner 1978; Kistner and Howard 1980).

In the present paper, the genus *Trichopsenius* is reported from China for the first time, represented by the new species *Trichopseniushuaxiensis* sp. nov., described and illustrated herein. All adults of the new species were collected in a nest of termites of the genus *Reticulitermes* from a flattened dead pine tree.

## ﻿Material and methods

Material examined is deposited in the Institute of Entomology, Guizhou University, Guiyang, China (**GUGC**).

Label data is quoted verbatim. The Chinese translation of each locality below the provincial level is included in parentheses at its first appearance in the text. Each type specimen bears the following label: ‘HOLOTYPE (red) (or PARATYPE (yellow)), m# (or f#), *Trichopseniushuaxiensis* sp. nov., Jiang, Huang & Chen, 2023.’.

Images of habitus and morphological details were taken using a Canon 5D Mark IV digital camera with a Mitutoyo Plan NIR 10 lens, a Godox MF12 flash was used as the light source, or a Nikon SMZ25 stereoscopic microscope with a Nikon DS-Ri2 camera. Zerene Stacker (ver. 1.04) was used for image stacking. Adobe Illustrator CS5 was used to prepare the line-drawings. All images were modified and grouped into plates in Adobe Photoshop CS5 Extended.

The following abbreviations are applied: **AL**—length of visible abdomen (in dorsal view) along the midline, **HL**—length of head from the anterior clypeal margin to the occipital constriction; **HW**—width of head across eyes; **PL**—length of pronotum along the midline; **PW**—maximum width of pronotum; **EL**—length of elytra along the suture; **EW**—maximum width of elytra; **BL**—Length of the body is a sum of PL + EL + AL.

## ﻿Taxonomy

### ﻿ Trichopsenius huaxiensis

Taxon classificationAnimaliaColeopteraStaphylinidae

Jiang, Huang & Chen sp. nov.

BAFF8024-F2BC-5B4F-9CF6-06F78F5719DC

https://zoobank.org/0D6CAF2E-7348-4216-85D5-88B47E9D920A

Figs 1 , 2 , 3

#### Type material.

**(10 exs: 7** ♂♂, **3** ♀♀) : ***Holotype***: China: ♂, labeled ‘China: Guizhou, Guiyang City (贵阳市), Huaxi District (花溪区), South Campus of Guizhou University (贵州大学南校区), Songlinpo (松林坡), 26°25'40"N, 106°40'06"E, H: 1105 m, 22.XI.2022, Jiang Ri-Xin leg.’ (GUGC). ***Paratypes***: 6 ♂♂, 1 ♀, with same label data as the holotype (GUGC); 2 ♀♀, with same label data as the holotype, except ‘23.XI.2022, Jiang Ri-Xin & Huang Xiu-Dong leg.’ (GUGC).

#### Diagnosis.

Body reddish brown with elytra and lateral lobe of tergite IX darker. Pronotum transverse with anterior margin M-shaped at middle. Surface of visible sternites and tergites finely covered with short setae and modified with a row of long setae at posterior margin. Median lobe of aedeagus with apical lobe knife shaped; left paramere with apical lobe thin and strongly sinuous; right paramere with apical lobe curved and weakly sinuous, with a thin seta at base.

*Trichopseniushuaxiensis* sp. nov. is most similar with *T.crassicornis* Naomi & Terayama, 1996; they share similar habitus characters, such as the sinuated anterior margin of the pronotum and the form of the antennomeres. The new species can be adequately distinguished from *T.crassicornis* by the following characters: 1) anterior margin of the head weakly curved (cf. straight in *T.crassicornis*); 2) median lobe of the aedeagus hemispherical at the base, apex without setae (cf. median lobe bulbous at base, apex with a short setae in *T.crassicornis*); and 3) apical lobe of the parameres much shorter, about 1/2 length of basal lobe in the right paramere, about 1/3 length of basal lobe in the left paramere (cf. apical lobe more than half length of basal lobe in both right and left parameres in *T.crassicornis*).

#### Description.

**Figure 1.**
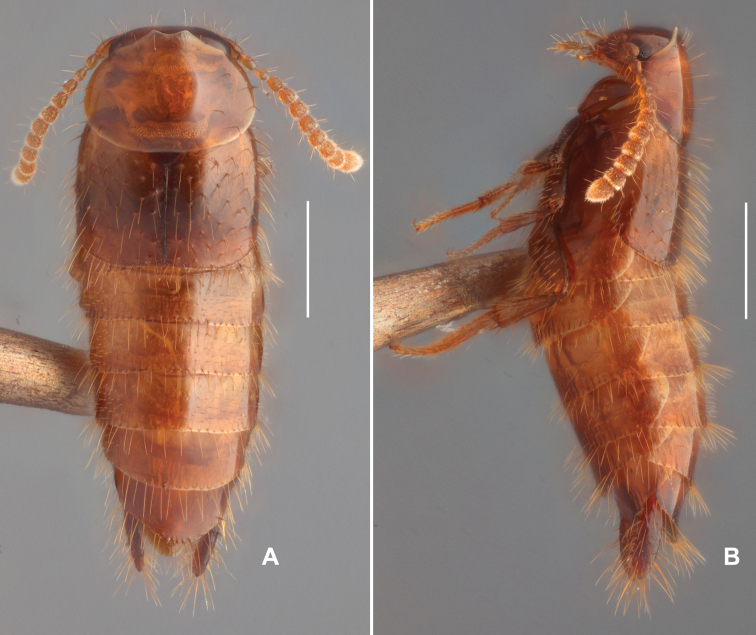
Habitus of *Trichopseniushuaxiensis* sp. nov., male **A** dorsal view **B** lateral view. Scale bars: 0.5 mm (**A, B**).

**Male. *Body*** (Fig. 1) glossy, reddish brown with elytra and lateral lobe of tergite IX slightly darker.

**Figure 2.**
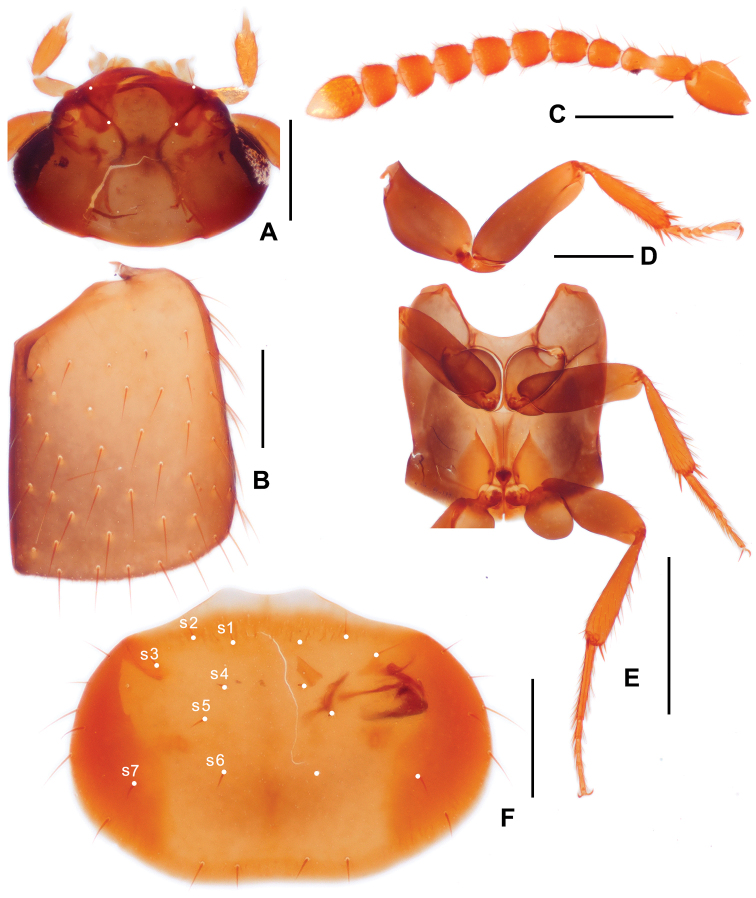
Diagnostic features of *Trichopseniushuaxiensis* sp. nov., male **A** head, dorsal view **B** elytra **C** antenna **D** front leg **E** meso- and metaventrites, with middle and hind legs **F** pronotum, dorsal view. Note: s1–s7, setae of pronotum. Scale bars: 0.2 mm (**A–D, F**); 0.5 mm (**E**).

***Head*** (Fig. 2A) transverse, widest across eyes, surface glossy, covered with sparse thin punctures, with a pair of long setae between eyes, and a pair of long setae located at middle of anterior margin. Antenna (Fig. 2C) 11-articled, antennomeres 1–3 shiny, only bearing sparse long setae, antennomere 4 covered with sparse long setae and several short setae, other antennomeres covered with dense short setae and sparse long setae. Antennomere 1 longer than wide, distinctly expanded at apical 1/2; antennomere 2 longer than wide, narrower than antennomere 1, expanded near middle; antennomere 3 slightly shorter than antennomere 2, longer than wide, widest near apex; antennomeres 4 and 5 similar in form, slightly longer that wide; antennomeres 6–10 similar, about as long as wide, near trapezoidal; antennomere 11 with apex moderately pointed, about 1.5 times as long as wide, widest at basal 2/5. Mandibles (Fig. 2A) simple and aduncous, apex cuspidal. Maxillary palpomere 3 (Fig. 2A) about twice as long as wide, palpomere 4 small and subulate.

***Pronotum*** (Figs 1, 2F) transverse, about 1.5 times as wide as long, widest around posterolateral corners, surface covered with sparse long setae and thin and shallow punctures. Anterior margin protruded, M-shaped at middle, with three pairs of long setae (Fig. 2F, s1–s3) present subapically. Lateral margins finely curved, with several long setae. Posterior margin near straight, slightly concaved at middle, with two pairs of long setae. Disc of pronotum with four pairs of long setae, three pairs (Fig. 2F, s4–s6) located near median line, one pair (Fig. 2F, s7) located near lateral margin.

***Elytron*** (Fig. 2B) longer than wide, with lateral margin near straight, and curved at posterior part, posterior margin curved. Surface of elytron finely covered with large punctures, each puncture bearing long setae.

***Meso- and metaventrites*** (Fig. 2E) covered with several short setae and pores laterally. Surface of mesoventrite modified with thin polygonal microsculpture, mesocoxae almost contiguous. Surface of metaventrite covered with thin and longitudinal microsculpture; median sulcus longitudinal and thin, extending from posterior margin to near anterior margin; metaventral plate sparsely covered with minute pores; hind trochanter large and near ovate, surface covered with sparse minute pores.

***Legs*** (Fig. 2D–E) simple, surface finely covered with short setae. Outer margins of tibiae covered with long setae, setae on inner margins much shorter than on out margin, and only present in apical half. Apex of tibiae with several spurs, two of them long and strong, others shorter and thinner. Surface of tarsus densely covered with long setae. Front tarsaomere 1 (Fig. 2D) slightly expended near apex, about as long as sum of length of tarsomeres 2 and 3; tarsomeres 2–4 similar in form, short; tarsomere 5 elongate, about as long as sum of tarsomeres 2–4. Middle tarsomere 1 (Fig. 2E) elongate, slightly shorter than sum of other four tarsomeres; tarsaomeres 2–4 similar in form, tarsaomere 2 longer than tarsaomeres 3 and 4; tarsaomere 5 elongate, shorter than sum of length of tarsaomeres 2–4. Hind tarsomere 1 strongly elongate, longer than sum of other four members; tarsomere 2 shorter than sum of tarsomeres 3–4; tarsomere 3 slightly longer than tarsomere 4; tarsomere 4 shortest; tarsomere 5 about as long as sum of length of tarsomeres 2–3.

**Figure 3.**
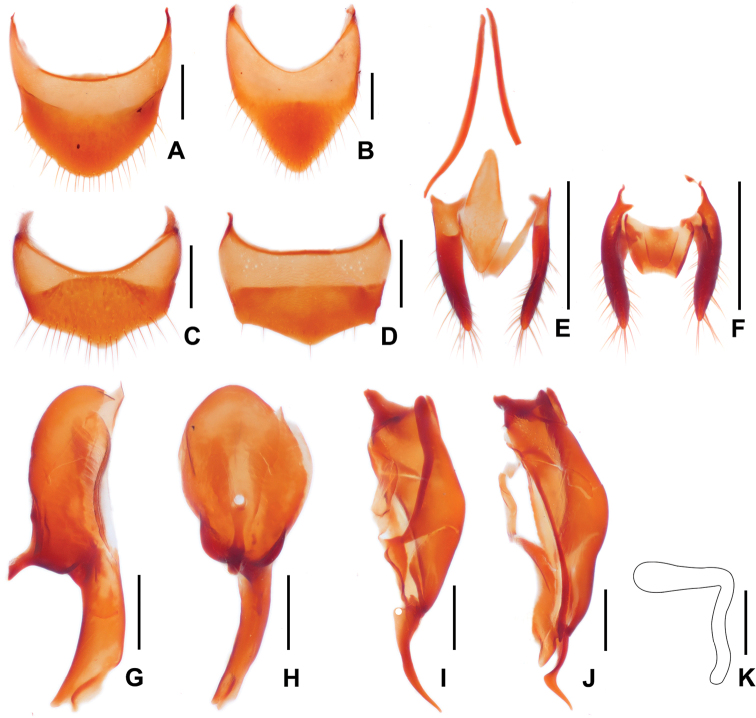
Diagnostic features of *Trichopseniushuaxiensis* sp. nov., **A, B, E, G–J** male; **C, D, F, K** female **A** tergite VIII, male **B** sternite VIII, male **C** tergite VIII, female **D** sternite VIII, female **E** tergite IX–X and sternite IX, male **F** ditto, female **G** median lobe of aedeagus, lateral view **H** ditto, ventral view **I** right paramere **J** left paramere **K** spermatheca. Scale bars: 0.05 mm (**K**); 0.2 mm (**A–D, G–J**); 0.5 mm in (**E, F**).

***Abdomen*** (Fig. 1A) gradually narrowed posteriorly, surface finely covered with short setae, all visible sternites and tergites modified with a row of long setae at posterior margin. Tergite VIII (Fig. 3A) about as long as wide, disc with several small pores and without setae, posterior margin rounded, margined by a row of long setae. Sternite VIII (Fig. 3B) longer than wide, disc with sparse small pores, without setae, posterior margin V-sharped, rounded at middle and margined by a row of long setae. Tergite IX (Fig. 3E) cylindrical, lobes long and slender, sparsely covered with setae of different lengths on posterior half; struts thin and long, finely curved, both ends narrower than middle part. Tergite X (Fig. 3E) membranous, inverted triangular, slightly concaved at middle of posterior margin. Sternite IX (Fig. 3E) rhombic, with middle of posterior margin with several short setae.

***Median lobe of aedeagus*** (Fig. 3G, H) with basal capsule longer than apical lobe; apical lobe knife shaped, with apex slightly expended, apex with a small zonal sclerite. Parameres (Fig. 3I, J) large, longer than median lobe, asymmetric. Left paramere (Fig. 3J) with apical lob shorter than in right paramere, thin and strongly sinuous, apex acute. Right paramere (Fig. 3I) with apical lobe curved and weakly sinuous, with a thin seta at base, apex acute.

***Measurements***: BL: 1.99–2.10 mm; HL: 0.30–0.35 mm, HW: 0.51–0.53 mm; PL: 0.38–0.40 mm, PW: 0.55–0.59 mm; EL: 0.41–0.44 mm; EW: 0.75–0.81 mm; AL: 1.20–1.26 mm.

**Female** (Fig. 3C, D, F, K), externally similar with male in habitus. Tergite VIII (Fig. 3C) transverse, disc with several small pores and sparsely covered with short setae; posterior margin curved with an apical rounded extension at middle, margined by a row of long setae. Sternite VIII (Fig. 3D) generally similar with tergite VIII in form, posterior margin with only several long setae. Tergite IX (Fig. 3F) cylindrical, lobes long and slender, sparsely covered with setae of different lengths on posterior half. Tergite X (Fig. 3F) membranous, inverted trapezoidal, slightly concaved at middle of posterior margin. Sternite IX (Fig. 3F) rhombic, with posterior margin rounded. Spermatheca (Fig. 3K) with apical and basal part near perpendicular to each other, basal part narrower than apical part, distinctly sinuated, apical part near straight, with apical expanded and round.

***Measurements***: BL: 1.98–2.10 mm; HL: 0.30–0.33 mm, HW: 0.50–0.55 mm; PL: 0.39–0.41 mm, PW: 0.55–0.58 mm; EL: 0.40–0.44 mm; EW: 0.78–0.82 mm; AL: 1.19–1.25 mm.

#### Distribution.

China (Guizhou).

#### Symbiotic host.

**Figure 4.**
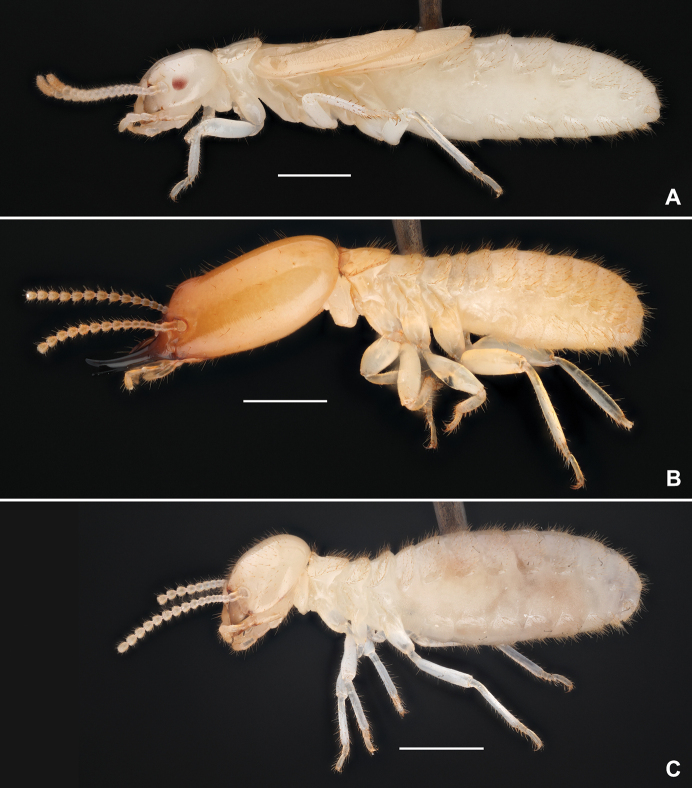
Host termite of *Trichopseniushuaxiensis* sp. nov., *Reticulitermes* sp. **A** neotenic reproductive **B** soldier **C** worker. Scale bars: 1 mm (**A–C**).

*Reticulitermes* sp. (Rhinotermitidae, Fig. 4).

#### Etymology.

The species is named after its type locality, Huaxi District (Guiyang City, Guizhou, China).

#### Biological notes.

**Figure 5.**
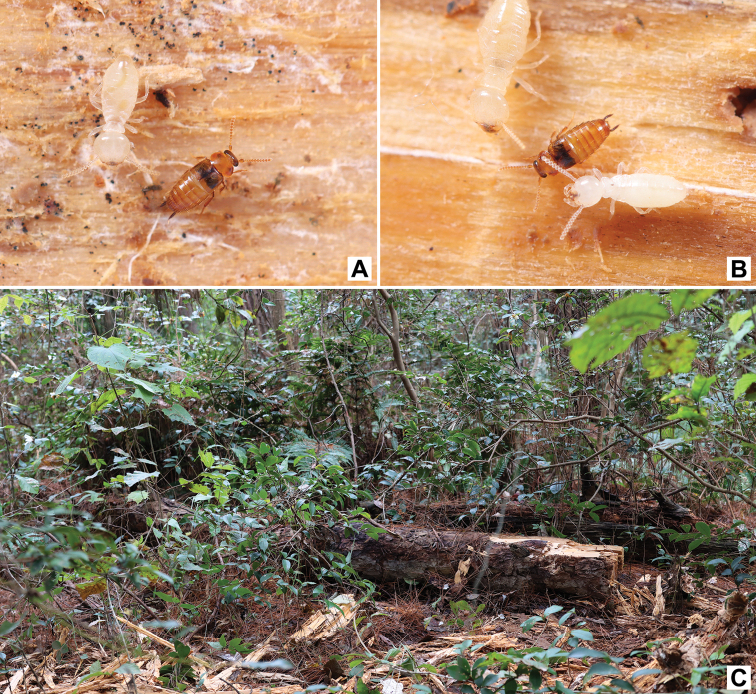
Habitat of *Trichopseniushuaxiensis* sp. nov. **A, B** adults with workers and larvae of *Reticulitermes* sp. **C** environment where *Trichopseniushuaxiensis* sp. nov. was found.

All adults of the new species were collected in November 2022, they were found in a dead and flattened pine tree where host termites were living (Fig. 5). There were obvious communications between the new species and its host termites after laboratory observation, including antennal touch at least. No attack behavior from their host termites was observed.

## Supplementary Material

XML Treatment for Trichopsenius huaxiensis
